# What is the role of family medicine in providing palliative care in Africa?

**DOI:** 10.4102/phcfm.v17i1.4879

**Published:** 2025-04-14

**Authors:** Jennie Morgan, Maggie de Swardt, Nuhamin Tekle Gebre, Mohja K.A. Marhoom, Edwina B. Opare-Lokko, Liz Gwyther

**Affiliations:** 1 Division of Family Medicine, Department of Family, Community and Emergency Care, Faculty of Health Sciences, University of Cape Town, Cape Town, South Africa; 2 Metro District Health, Western Cape Department of Health and Wellness, Cape Town, South Africa; 3 Division of Interdisciplinary Palliative Care and Medicine, Department of Family, Community and Emergency care, Faculty of Health Sciences, University of Cape Town, Cape Town, South Africa; 4 Cicely Saunders Institute of Palliative Care, Rehabilitation and Policy, Faculty of Nursing, Midwifery and Palliative Care, King’s College London, London, United Kingdom; 5 Ministry of Health, Ethiopia; 6 Nursing and Palliative Care Departments, Palliative Care Unit, Comboni College of Science and Technology, Khartoum Oncology Hospital, Sudan; 7 Faculty of Family Medicine, Ghana College of Physicians and Surgeons, Accra, Ghana; 8 Department of Family Medicine, Greater Accra Regional Hospital, Accra, Ghana

**Keywords:** family medicine, palliative care, family physician, undergraduate training, postgraduate training

## Abstract

Although palliative care is known to effectively relieve serious health-related suffering (SHS), it is not yet widely available, particularly in Africa. Primary health care has been recognised as an effective means to enhance access to palliative care and achieve universal health coverage. As family physicians play an important role in the delivery of primary health care, this article seeks to illustrate how the family medicine speciality is contributing to efforts to ensure palliative care services are provided throughout the African continent. The World Health Organization recommends three tiers of training for healthcare providers to enhance competencies in palliative care. This training has played out differently in various African countries. This article focuses on the countries represented by the authors, namely South Africa, Ethiopia, Sudan and Ghana. In providing continuous, coordinated, holistic care to patients along the life course, family physicians can anticipate and relieve suffering in a timely manner in ways that no other specialities have been trained to do. We propose that all family physicians’ training programmes in Africa prioritise palliative care training along with other leading clinical areas, to ensure that the significant numbers of people dying from SHS receive holistic care and die with dignity.

## Introduction

It is estimated that by 2060, 47% of all deaths globally will be attributed to illnesses associated with serious health-related suffering (SHS) such as cardiovascular diseases, dementia and cancer.^1^ This is an absolute increase of 87%, and the vast majority of global health-related suffering (83%) will occur in low to middle income countries.^1^ Although palliative care is known to effectively relieve SHS, and the World Health Organization (WHO) endorsed Resolution 67.19 calling for its integration into healthcare systems and health professional education, it is not yet widely available, particularly in Africa.^2,3^

Primary health care has been recognised as an effective means to enhance access to palliative care and achieve universal health coverage.^4^ As family physicians play an important role in the delivery of primary health care, this article seeks to illustrate how the family medicine speciality is contributing to efforts to ensure palliative care services are provided throughout the African continent.

The principles of family medicine and palliative care exhibit significant overlap as both adopt a person-centred, family-orientated, biopsychosocial, and spiritual approach to care throughout the life course regardless of age, gender, diagnosis or prognosis. The emphasis on clear communication, thorough clinical examination and a collaborative team-based approach, essential for addressing palliative care needs, aligns closely with the foundational principles of family medicine.^5,6^

Family physicians are best placed to lead clinical services, teams, and advocacy efforts in palliative care.^7^ With the growing demand for palliative care across Africa, effective leadership is crucial to drive policy changes, deliver training, and provide expert clinical support when challenges arise. Positioned within district hospitals or communities, family physicians can ensure patients receive care closer to home, avoiding distant hospitalisations. Their role includes guiding patients towards appropriate care that prioritises quality of life and respects individual preferences, while minimising unnecessary, costly interventions. This leadership fosters a patient-centred approach to end-of-life care within community and healthcare settings.

Primary care functions as the first point of contact for individuals seeking healthcare services within the community. It plays a critical role in maintaining continuity of care, facilitating efficient communication, and encouraging active community participation. Through these mechanisms, primary care enables individuals to assess and address their own palliative care needs, ensuring timely intervention and reducing delays in care.^4^

Furthermore, family physicians bridge gaps between primary and specialised care, advocate for resources, and implement culturally sensitive practices. Equipping these generalists with palliative care skills enables healthcare systems to improve the reach and quality of care, particularly in underserved African regions.^8^

Family physicians, by nature of the level at which they optimally function in the healthcare system, should be the most accessible specialists in Africa. They are uniquely positioned to provide basic and intermediate palliative care in all areas of the continent. Family physicians are still small in number and not as widely available as they could be. Through their networks, they can reach many people and can supervise many healthcare workers (HCWs). They need the skills to provide and to support others in their provision of palliative care.

## Service delivery

In many areas of Africa, specialists are in the minority. All HCWs need an understanding of palliative care, and they should have basic palliative care training.^2^ Healthcare workers with no additional postgraduate training are providing the majority of primary care. Thus, basic palliative care needs to be taught in every area of undergraduate HCW training.^2^ This allows all HCWs to manage less complex palliative care patients and to understand when to consult about more complex cases. We need to ask, ‘who do they ask for help when palliative care is not yet a recognised speciality in Africa? Who is best placed to manage the needs of the more complex palliative care patients?’

The WHO recommends three tiers of training for HCWs to enhance competencies in palliative care: (1) basic palliative care training for all HCWs; (2) intermediate-level training for healthcare professionals who frequently encounter palliative care patients, although it is not their primary clinical focus; and (3) specialist-level training for healthcare professionals dedicated exclusively to palliative care.^2^ From this description, it is clear that all family physicians should have intermediate level of palliative care training and should be able to upskill other HCWs to gain a basic level of palliative care training.

This has played out differently in various African countries. This article focuses on the countries represented by the authors. Table 1 summarises the current palliative care training in the four countries represented by the authors. For example, in Ghana, family medicine has led palliative care development through education and service provision since 2011, setting up units in four tertiary level hospitals.^9^

**TABLE 1:** Training status across four African countries.

Country	Undergraduate training	Postgraduate training	Initiatives and progress
South Africa	Basic training varied (6–46 h).^13^	Includes palliative care as one of the 22 entrustable professional activities (EPA), emphasis on basic and intermediate care.^14^	Ongoing efforts to standardise undergraduate training; EPA integration Accreditation of Subspeciality in Palliative Medicine in process
Sudan^15^	Integration in undergraduate nursing curriculum.	No formal postgraduate module; efforts underway for healthcare professionals.	Introduction of palliative care courses
Ghana	Integration in undergraduate nursing curriculum. Medical schools: variable basic palliative care content in 3 schools. (4 h–2 weeks)	Palliative Medicine Fellowship for doctors^16^; 3-year Palliative Care residency for nurses.^17^ Short basic and advanced postgraduate certificate courses available.^18^ Family Medicine residents, complete mandatory rotation (2–4 weeks). Elective rotation for oncology and anaesthesia residents. Basic and intermediate curriculum development for General and Children’s Palliative Care underway.	Leadership in national strategy. Active roles in professional associations
Ethiopia	Integration in undergraduate nursing curricula.	In-service short courses; ongoing efforts to formally incorporate the approach in other postgraduate programmes.	Coordination in training and advocacy

Family physicians are fast becoming leaders and advocates for our vulnerable patients.^7^ In Ethiopia, they are advocating for and making significant efforts towards the integration of palliative care at all levels of care, with particular emphasis on home-based palliative care. Such efforts include informing policy strategies through research, training multidisciplinary HCWs, empowering communities through information and repurposing existing community structures to support people with life-threatening illnesses and their families as well as navigating ways to improve access to essential medications such as morphine.^10,11^

The absence of a formal palliative care framework in some countries, for example Sudan, means that many patients suffering from chronic and terminal conditions do not receive the compassionate, holistic care that palliative care offers. This lack of recognition often results in fragmented care, with patients being referred to general healthcare providers who may lack the specialised skills needed for effective palliative management.^12^

## Advocacy and lessons learned in palliative care recognition

Across Africa, significant strides have been made through partnerships between family medicine and palliative care advocates, policymakers, and regulatory authorities. In South Africa, advocacy efforts through the Health Professions Council of South Africa (HPCSA) have approved a new sub-speciality in palliative medicine (MPhil PM) within the framework of family medicine. This initiative will not only enhance training but also formalise recognition and integration of palliative care into healthcare systems. Advocacy within the Colleges of Medicine of South Africa has resulted in five specialities planning to enrol fellows in the new MPhil fellowship course.

In Ghana, family physicians played a pivotal role in establishing the Palliative Medicine Fellowship and contributing to national policy frameworks. Their involvement in curriculum development and research has strengthened palliative care education and service delivery across multiple disciplines.

Sudan and Ethiopia are making progress in integrating palliative care into training curricula.

Reflecting back on the success of these initiatives, a pan-African approach involving sustained engagement with policymakers, structured training, alignment with existing healthcare frameworks and interdisciplinary collaboration are essential to strengthen education, policy, and service delivery as well as accelerate progress.

## Conclusion

The superpower of combining palliative medicine with family medicine lies in the fact that our multifaceted training gives us unique insights into the patient’s illness journey and their family dynamics. In providing continuous, coordinated, holistic care to patients along the life course,^6^ family physicians can anticipate and relieve suffering in a timely manner in ways that no other specialities have been trained to do. We are connected to and unify all the specialities, thus, perfectly positioned to lead the way in palliative medicine in a continent where there are few specialists. We propose that all family physician training programmes in Africa prioritise palliative care training along with other leading clinical areas, to ensure that the significant numbers of people dying from SHS receive holistic care and die with dignity.
